# High Prevalence of Methicillin-Resistant *Staphylococcus aureus* among Patients with Septic Arthritis Caused by *Staphylococcus aureus*

**DOI:** 10.1371/journal.pone.0127150

**Published:** 2015-05-21

**Authors:** Wei-Ting Lin, Chung-Da Wu, Shun-Chien Cheng, Chong-Chi Chiu, Chi-Chou Tseng, Huan-Tee Chan, Po-Yih Chen, Chien-Ming Chao

**Affiliations:** 1 Department of Orthopaedics, Chi Mei Medical Center, Tainan, Taiwan; 2 Department of Physical Therapy, Shu Zen Junior College of Medicine and Management, Tainan, Taiwan; 3 Department of General Surgery, Chi Mei Medical Center, Liouying, Tainan, Taiwan; 4 Department of Electrical Engineering, Southern Taiwan University of Science and Technology, Tainan, Taiwan; 5 Department of Orthopaedics, Chi Mei Medical Center, Liouying, Tainan, Taiwan; 6 Department of Intensive Care Medicine, Chi Mei Medical Center, Liouying, Tainan, Taiwan; 7 Department of Nursing, Min-Hwei College of Health Care Management, Tainan, Taiwan; Rockefeller University, UNITED STATES

## Abstract

**Background:**

This study investigated the clinical characteristics of patients with septic arthritis caused by *Staphylococcus aureus* and tried to identify the risk factors for methicillin-resistant *S*. *aureus* (MRSA) arthritis.

**Methods:**

Between January 2008 and December 2011, patients with septic arthritis caused by *S*. *aureus* were identified from the computerized databases of a regional hospital and a medical center in southern Taiwan. The medical records of these patients were retrospectively reviewed.

**Results:**

A total of 93 patients with *S*. *aureus* arthritis were identified, and MRSA arthritis was found in 38 (40.9%) cases. The mean age of the patients was 58 years, and 86 (92.5%) episodes were classified as community-acquired infections. Diabetes mellitus (n = 41, 44.1%) was the most common underlying disease, followed by chronic kidney disease and liver cirrhosis. Patients with MRSA arthritis were more frequently elderly and found in the setting of healthcare-associated infection than patients with methicillin-susceptible *S*. *aureus* (MSSA) infections. No other significant differences in clinical manifestations and outcomes were noted between these two groups of patients. Overall, the in-hospital mortality rate was 5.4%, and diabetes mellitus was the only risk factor for mortality.

**Conclusions:**

MRSA is emerging in the setting of community-acquired septic arthritis. MRSA septic arthritis is more likely to develop in the elderly and in healthcare-associated infections than MSSA septic arthritis.

## Background

Septic arthritis is not an uncommon disease, and its yearly incidence ranges from 2 to 10 per 100,000 patients [1–4]. *Staphylococcus aureus* and *Streptococcus* spp. are the most common pathogens causing septic arthritis [5–7]. In fact, the emergence of methicillin-resistant *S*. *aureus* (MRSA) is a growing clinical challenge in hospital-associated settings as well as in community settings in the United States and around the world. [8]. In Taiwan, MRSA infection accounts for about 60% of nosocomial *S*. *aureus* infections [9] and it is also an emerging community-acquired pathogen [10]. Although there have been several studies about MRSA arthritis in western countries [7,11–17], the data of Asian is still limited. In a study of 51 adults in Taiwan with culture-proven community-onset septic arthritis, *S*. *aureus* was the most common etiology (n = 30, 58.9%) [16]. MRSA comprised 22% of *S*. *aureus* arthritis cases in a recent retrospective study in Japan [17]. Both of the above studies indicate the clinical significance of *S*. *aureus*, including MRSA, in septic arthritis. Therefore, we performed this study to investigate the clinical manifestations of MRSA arthritis and compare them with those of methicillin-susceptible *S*. *aureus* (MSSA). Additionally, we wanted to identify risk factors for MRSA arthritis and analyze the prognostic factors of mortality among patients with *S*. *aureus* arthritis.

## Methods

### Hospital setting and patient selection

This study design is a clinical case-series and was conducted at two hospitals, Chi Mei Medical Center, a 1290-bed referral medical center, and Chi Mei Medical Center, Liouying branch, a 900-bed regional hospital located in Tainan, Taiwan. All patients with culture-confirmed *S*. *aureus* arthritis were identified from the computerized databases of the hospitals between January 2008 and December 2011. The medical records of all patients were retrospectively reviewed. The following patient characteristics and laboratory findings were collected from the records: age, gender, underlying conditions (history of chemotherapy agent use, diabetes mellitus, liver cirrhosis, end-stage renal disease, and active cancer), laboratory data, microbiological findings, antimicrobial susceptibility test results, and patient outcome. The records and information of patients were anonymized and de-identified prior to analysis. Therefore, informed consent was not required and was specifically waived by the Institutional Review Board. Ethics approval was obtained from the Institution Review Board of Chi Mei Medical Center in accordance with the 1964 ethical standards for medical research involving human subjects of the Declaration of Helsinki as revised in 2000.

### Bacterial isolates and antimicrobial susceptibilities

Identification of *S*. *aureus* isolates was achieved by standard techniques. All of the isolates were determined to be Gram-positive cocci with β-hemolysis on 5% sheep blood agar and gave positive results on catalase, coagulase, DNase, and mannitol fermentation tests. Susceptibilities of these isolates to a battery of antimicrobial agents, including gentamicin, minocycline, oxacillin, teicoplanin, augmentin, tigecycline, clindamycin, penicillin, fucidic acid, and trimethoprim-sulfamethoxazole, were determined using the disk diffusion method as described by the Clinical and Laboratory Standards Institute [18].

### Definitions

Healthcare-associated infections were defined as those which were acquired during the course of treatment for other conditions within a healthcare setting [19]. Leukocytosis was defined as a white blood cell count > 11,000/mL. A standard definition for polymicrobial infections was isolation of other pathogens from synovial fluid, in addition to *S*. *aureus*. In-hospital mortality was defined as death due to any cause during hospitalization. Immunocompromised status was defined if patients had liver cirrhosis, diabetes mellitus, end-stage renal disease, or active cancer or were receiving chemotherapy agents. Inappropriate use of empirical antibiotics was considered as the empirical usage of antimicrobial agents that were ineffective in vitro against *S*. *aureus* isolates.

### Statistical analysis

Continuous variables were expressed as mean ± standard deviation. These variables were compared using the Wilcoxon rank sum test or Student’s independent *t* test, as appropriate. Categorical variables were compared using the chi-square test or Fisher’s exact test. Patient outcomes for in-hospital mortality were analyzed using the chi-square test. A *P* value < 0.05 was considered to represent statistical significance. All statistical analyses were conducted using the statistical package SPSS for Windows (Version 11.0, SPSS, Chicago, Il, USA).

## Results

### Clinical characteristics

During the study period, a total of 194 cases of septic arthritis were identified and 93 (47.9%) cases were caused by *S*. *aureus*. The clinical characteristics of the 93 patients with septic arthritis caused by *S*. *aureus* are summarized in Table 1. The patients ranged in age from 3 to 91 years (median, 58 years, interquartile range, 17 years). Most of the patients were men (n = 67, 63%), and more than 90% of the infections were classified as community-acquired. Diabetes mellitus (n = 41, 44.1%) was the most common underlying disease, followed by chronic kidney disease and liver cirrhosis. Seven patients had underlying malignancy, including hepatoma (n = 3), lymphoma (n = 2), esophageal cancer (n = 1), and skin cancer (n = 1). One patient had bullous pemphigoid. Overall, more than 60% of patients were considered immunocompromised, and about one-forth of patients had a history of trauma before acquiring septic arthritis. The most common site was the knee, followed by the hip. Two patients had multiple joint involvement, one in the lumbar spine and hip, and the other in the elbow and hip. Forty-eight patients had leukocytosis. C-reactive protein levels > 6 mg/L were found in 70 of the 72 (97.2%) patients with available results. Polymicrobial infections were observed in three patients, with *Chryseomonas luteola*, *Pseudomonas aeruginosa*, and *Streptococcus pyogens* respectively. Twelve patients had positive Gram stains for Gram positive cocci in examinations of the synovial fluid. Twenty-eight (30.1%) patients had concurrent *S*. *aureus* bacteremia. All 38 patients receiving inappropriate empirical antibiotic treatment belonged to the MRSA arthritis group.

**Table 1:** Clinical characteristic of 93 patients with septic arthritis caused by *Staphylococcus aureus*.

	No (%) of patients(n = 93)
Median age, years (interquartile range)	58 (17)
Age ≥ 65 years, n (%)	38 (40.9)
Male, n (%)	67 (63.4)
Community-acquired infection	86 (92.5)
Underlying condition	
Diabetes mellitus	41 (44.1)
Chronic kidney disease	25 (26.9)
Liver cirrhosis	12 (12.9)
Gout arthritis	12 (12.9)
Active cancer	7 (7.5)
Use of steroid*	4 (4.3)
Bullous pemphigoid	1 (1.1)
Immunocompromised status	59 (63.4)
Trauma related	22 (23.7)
Type of joint	
Native joint	70 (75.3)
Prosthesis	23 (24.7)
Involved joint	
Knee	51 (54.8)
Hip	23 (24.7)
Elbow	9 (9.7)
Shoulder	5 (5.4)
Ankle	4 (4.3)
Spine	2 (2.2)
Toe	1 (1.1)
Multiple joint involvement	2 (2.2)
Laboratory findings	
White blood cell (cell/uL)	12505.3 ± 5937.3
Hemoglobin (g/dL)	11.6 ± 2.2
Platelet (cell/uL)	288428.6 ± 151379.4
ESR (mg/dL) (n = 65)	73.7 ± 37.2
C-reactive protein (mg/L) (n = 72)	95.5 ± 84.5
Polymicrobial infections	3 (3.2)
Findings of synovial fluid	
White blood cell (cell/uL)	77268.4 ± 85675.5
Positive Gram stain (%)	12 (12.9)
Bacteremia	28 (30.1)
Drainage	2 (2.2)
Inappropriate use of empirical antibiotics	38 (40.9)
Surgery treatment	78 (83.9)
Mortality	5 (5.4)

*Use of steroid was defined as use of ≧ 20 mg per day of prednisone for more than two weeks.

### Comparison between patients with MSSA arthritis and MRSA arthritis

We compared the clinical manifestations between 55 patients with MSSA arthritis and 38 patients with MRSA arthritis (Table 2). Patients with MRSA were more frequently > 65 years old than patients with MSSA. In contrast, all patients with MSSA had community-acquired infection and the frequency of healthcare-associated infections was significantly less than in patients with MRSA. No other significant differences, including age, sex, type of infection, underlying conditions, cause of arthritis, clinical manifestations, treatment, and outcome, were noted between the two groups. Four patients with MRSA had received antibiotics prior to the episode of septic arthritis. Three of the patients were given 3^rd^ generation cephalosporins and one was given quinolones.

**Table 2:** Comparison of 55 and 38 patients with septic arthritis caused by methicillin-susceptible *Staphylococcus aureus* and methicillin-resistant *Staphylococcus aureus*.

	No (%) of patients with methicillin-susceptible *Staphylococcus aureus*(n = 55)	No (%) of patients with methicillin-resistant *Staphylococcus aureus*(n = 38)	P value
Age ≥ 65 years, n (%)	17 (30.9)	21 (55.3)	0.03
Male, n (%)	41 (74.5)	26 (68.4)	0.69
Community-acquired infection	55 (100.0)	31 (81.6)	0.04
Underlying condition			
Diabetes mellitus	27 (49.1)	14 (36.8)	0.34
Chronic kidney disease	14 (25.5)	11 (28.9)	0.27
Liver cirrhosis	8 (14.5)	4 (10.5)	0.81
Gout arthritis	7 (12.7)	12 (12.9)	0.77
Active cancer	5 (9.1)	2 (5.3)	0.78
Use of steroid*	2 (3.6)	2 (5.3)	0.90
Bullous pemphigoid	0 (0.0)	1 (2.6)	0.88
Immunocompromised status	36 (65.5)	23 (60.5)	0.79
Trauma related	11 (20.0)	11 (28.9)	0.46
Type of joint			0.14
Native joint	45 (81.8)	25 (65.8)	
Prosthesis	10 (18.2)	13 (34.2)	
Polymicrobial infections	3 (5.5)	0 (0.0)	0.39
Previous use of antibiotic	0 (0.0)	4 (10.5)	0.05
Positive Gram stain in synovial fluid	7 (12.7)	5 (13.2)	0.81
Bacteremia	18 (32.7)	10 (26.3)	0.67
Drainage	2 (3.6)	0 (0.0)	0.66
Surgery treatment	44 (80.0)	34 (89.5)	0.35
Mortality	3 (5.5)	2 (5.3)	0.67

*Use of steroid was defined as use of ≧ 20 mg per day of prednisone for more than two weeks.

### Outcome analysis

Overall, there were five deaths in this series. All of these patients were immunocompromised and had diabetes mellitus. Three cases were caused by MSSA and the other two by MRSA. Three of these patients were > 65 years and three had concomitant bacteremia. The only risk factor for mortality was diabetes mellitus (Table 3). Overall, 9 patients were admitted to the intensive care unit and 4 patients had acute respiratory failure. The overall in-hospital mortality was 5.4%.

**Table 3:** Risk factors of mortality of patients with septic arthritis caused by *Staphylococcus aureus*.

Characteristics	Survivor(n = 88)	Mortality(n = 5)	*P* value
Elderly (age> 65 years)	35 (39.8)	3 (60.0)	0.67
Male	63 (71.6)	4 (80.0)	0.92
Diabetes mellitus	36 (40.9)	5 (100.0)	0.03
Chronic kidney disease	23 (26.1)	2 (40.0)	0.87
Liver cirrhosis	11 (12.5)	1 (20.0)	0.84
Prosthesis	22 (25.2)	1 (20.0)	0.79
Leukocytosis	46 (52.3)	2 (40.0)	0.94
MRSA	36 (40.9)	2 (40.0)	0.67
Polymicrobial infection	3 (3.4)	0 (0.0)	0.38
Bacteremia	25 (28.4)	3 (60.0)	0.32
Inappropriate use of empirical antibiotic	36 (40.9)	2 (40.0)	0.67
Community-acquired infection	81 (92.0)	5 (100.0)	0.83

## Discussion

This study enrolled 93 patients with *S*. *aureus* arthritis and provided several useful findings in this clinical setting. Most important, about 40% of cases of *S*. *aureus* arthritis were caused by MRSA, and more than 80% of MRSA arthritis cases were classified as community-acquired infections. Ross found only 15 of 59 (25%) septic arthritis cases in the USA involved MRSA [12]. Clerc investigated 233 episodes of septic arthritis in Switzerland between 1999 and 2008 and showed that *S*. *aureus* caused 115 episodes, of which only 11 (9.6%) were caused by MRSA [14]. Another study of 58 adult patients in the UK with hematogenous septic arthritis from June 2000 to June 2005 revealed that 15 (25.9%) clinical isolates were methicillin-resistant [13]. In a retrospective analysis of 53 adults with septic arthritis in Japan from 1955 to 2005, 22% of *S*. *aureus* arthritis cases were caused by MRSA [17]. In contrast, the prevalence of MRSA in *S*. *aureus* arthritis in the present work was much higher than in previous studies [12–14, 17]. This is consistent with previous findings about different types of *S*. *aureus* infection in Taiwan, in which MRSA infection accounted for more than half of nosocomial *S*. *aureus* infections [9] and was an emerging pathogen causing community-acquired infections [10]. Although the differences may be due to the different study populations and the fact that the epidemiology of MRSA may vary geographically, our findings suggest that MRSA should be seriously considered as one of the pathogens causing septic arthritis in this era of increasing antibiotic resistance, even in the setting of community-acquired infections. To manage the threat of MRSA infections in Taiwan, the importance of infection control measures, including hand hygiene, contact isolation and use of antibiotics, should be emphasized.

We further tried to find the risk factors for MRSA arthritis among patients with *S*. *aureus* arthritis. Overall, we found that age > 65 years was the only one risk factor for MRSA arthritis in the present work. This is consistent with previous studies. Ross found that patients with MRSA arthritis were significantly older than patients with non-MRSA arthritis (69 vs. 54, p = 0.003) [12]. Al-Nammari also found that MRSA patients were older than non-MRSA patients with a mean age of 76 versus 44 years (p < 0.05) [13]. Moreover, we noted another significant factor—healthcare-associated infections—in this study. MRSA arthritis more frequently developed in the setting of healthcare-associated infection. This finding is consistent with a previous study [12]. In summary, MRSA should be considered in the differential diagnosis of septic arthritis, especially in the elderly population and in healthcare-associated infections.

The in-hospital mortality in this study was only 5.4%, with no significant difference between patients infected with MSSA and MRSA. Overall, these outcomes were relatively favorable compared with previous studies in which mortality rates varied from 13.3% to 20% [12,13]. The difference may be due to different study populations or different study designs. In addition, the five patients who died all had underlying diabetes mellitus, and diabetes mellitus was found to be the only significant poor prognostic factor. In contrast to other studies, we did not find that inappropriate empirical antibiotics were associated with a poor outcome [13].

In the present work, most of the patients had a final diagnosis of *S*. *aureus* arthritis after positive culture results from synovial fluid. Initial Gram stain examinations of the joint fluid showed Gram positive cocci in clusters in only twelve (12.9%) patients.

This study had several limitations. First, the sample size in this retrospective study may have been too small to perform meaningful risk factor analysis for mortality. Second, there were no available isolates clinically for molecular investigation, whether to study their genetic diversity, to determine clonal or genetic diversity, or to determine their classification of typical HA-MRSA or CA-MRSA clones. Although we had tried to investigate the epidemiologic data and establish the resistance profile of the tissue isolates—clonal spread, we did not make any progress. Third, the study was performed in two hospitals in southern Taiwan, and the conditions may not represent all *S*. *aureus* arthritis in Taiwan. Finally, we used all-cause mortality for outcome analysis and did not evaluate mortality attributable to septic arthritis.

In conclusion, this study in Taiwan showed a high prevalence of MRSA in patients with septic arthritis, even in community-acquired infections. Moreover, MRSA arthritis may be more likely to develop in elderly patients and in healthcare-associated infections than MSSA. Finally, diabetes mellitus was found to be associated with mortality in patients with *S*. *aureus* arthritis.

## References

[1] Mathews CJ, Weston VC, Jones A, Field M, Coarkley G. Bacterial septic arthritis in adults. Lancet 2010;375: 846–855. 10.1016/S0140-6736(09)61595-6 20206778

[2] Shirtliff ME, Mader JT. Acute septic arthritis. Clin Microbiol Rev 2002;15: 527–544. 12364368 10.1128/CMR.15.4.527-544.2002 PMC126863

[3] Pioro MH, Mandell BF. Septic arthritis. Rheum Dis Clin North Am 1997;23: 239–258. 9156391 10.1016/s0889-857x(05)70328-8

[4] Ryan MJ, Kavanagh R, Wall PG, Hazleman BL. Bacterial joint infections in England and Wales: analyses of bacterial isolates over a four year period. British J Rheum 1997;36: 370–373. 10.1093/rheumatology/36.3.370 9133971

[5] Mathews CJ, Coakley G. Septic arthritis: current diagnostic and therapeutic algorithm. Curr Opin Rheumatol 2008;20: 457–462. 10.1097/BOR.0b013e3283036975 18525361

[6] Goldenberg DL, Reed JL. Bacterial arthritis. N Engl J Med 1985;312: 764–771. 3883171 10.1056/NEJM198503213121206

[7] Frazee BW, Fee C, Lambert L. How come is MRSA in adult septic arthritis. Ann Emerg Med 2009;54: 695–700. 10.1016/j.annemergmed.2009.06.511 19665261

[8] Boucher HW, Corey GR. Epidemiology of methicillin-resistant *Staphylococcus aureus* . Clin Infect Dis 2008;46: S344–349. 18462089 10.1086/533590

[9] Hsueh PR, Chen ML, Sun CC, Chen WH, Pan HJ, Yang LS, et al Antimicrobial drug resistance in pathogens causing nosocomial infections at a university hospital in Taiwan, 1981–1999. Emerg Infect Dis 2002;8: 63–68. 11749750 10.3201/eid0801.000454 PMC2730256

[10] Chen CJ, Huang YC. Community-acquired methicillin-resistant *Staphylococcus aureus* in Taiwan. J Microbiol Immunol Infect 2005;38:376–382. 16341337

[11] Gupta MN, Sturrock RD, Field M. Prospective comparative study of patients with culture proven and high suspicion of adult onset septic arthritis. Ann Rheum Dis 2003;62: 327–331. 12634231 10.1136/ard.62.4.327 PMC1754487

[12] Ross JJ, Davidson L. Methicillin-resistant *Staphylococcus aureus* septic arthritis: an emerging clinical syndrome. Rheumatology 2005;44: 1197–1198. 16049047 10.1093/rheumatology/kei035

[13] Al-Nammari SS, Babak P, Venkatesh R. Methicillin resistant *Staphylococcus aureus* versus methicillin sensitive *Staphylococcus aureus* adult haematologenous septic arthritis. Arch Ortho Trauma Surg 2007;127: 537–542. 17260151 10.1007/s00402-007-0285-z

[14] Clerc O, Prod’hom G, Greub G, Zanetti G, Senn L. Adult native septic arthritis: a review of 10 years of experience and lesions for empirical antibiotic therapy. J Antimicrob Chemother 2011,; 66: 1168–1173. 10.1093/jac/dkr047 21393124

[15] Byrne PA, Hosein IK, Camilleri J. Methicillin-resistant *Staphylococcus aureus* septic arthritis: urgent and emergent. Clin Rheumatol 1998;17: 407–408. 9805190 10.1007/BF01450904

[16] Chuang YC, Wang JL, Chen YC, Chang SC. Characteristics and outcomes of community-onset septic arthritis. J Microbiol Immunol Infect 2009;42: 258–264. 19812860

[17] Okano T, Enokida M, Otsuki R, Teshima R. Recent trends in adult-onset septic arthritis of the knee and hip: retrospective analysis of patients treated during the past 50 years. J Infect Chemother 2011;17: 666–670. 21584725 10.1007/s10156-011-0244-z

[18] Clinical and Laboratory Standards Institute. Performance standards for antimicrobial susceptibility testing. Nineteenth informational supplement. M100-S20. Wayne, PA: CLSI, 2010.

[19] Horan TC, Andrus M, Dudec MA. CDC/NHSN surveillance definition of health care-associated infection in the acute care setting. Am J Infect Control 2008;36: 309–332. 10.1016/j.ajic.2008.03.002 18538699

